# Recent Advances and Future Perspectives in the Development of Therapeutic Approaches for Neurodegenerative Diseases

**DOI:** 10.3390/brainsci10090633

**Published:** 2020-09-11

**Authors:** Melissa Bowerman

**Affiliations:** 1 School of Medicine, Keele University, Staffordshire ST5 5BG, UK; m.bowerman@keele.ac.uk; 2 Wolfson Centre for Inherited Neuromuscular Disease, RJAH Orthopaedic Hospital, Oswestry SY10 7AG, UK

**Keywords:** neurodegenerative diseases, Alzheimer’s disease (AD), Parkinson’s disease (PD), Huntington’s disease (HD), brain, therapy

Neurodegenerative diseases such as Alzheimer’s disease (AD), Parkinson’s disease (PD) and Huntington’s disease (HD), severely impact the function of neuronal cells in the brain and have devastating consequences on the quality of life of patients and their families [1,2,3]. In their 2018 review, Hussain et al. presented common mechanistic pathologies that contribute to the neurodegenerative processes in these conditions and discussed novel therapeutic approaches aimed at targeting these aberrant signaling cascades [4]. Presently, the development of new treatment strategies for AD, PD and HD remain at the pre-clinical and clinical stages, while commercially available and approved drugs predominantly alleviate symptoms temporarily without significantly altering disease progression [5,6,7]. Notably, the most commonly prescribed treatments for AD (memantine) and PD (levodopa) were approved by the U.S. Food and Drug Administration (FDA) in the early 2000s and 1970s [5,8], respectively, and no new life-changing disease-modifying drugs have reached patients since. The first regulated treatment for HD appeared in the late 2000s (tetrabenazine) and remained the sole option until a chemically modified version of the drug (deutetrabenazine) was recently approved by the FDA. Thus, despite herculean efforts from scientists, clinicians and pharmacological companies, we remain in a state of urgent need for groundbreaking therapies for neurodegenerative diseases.

In their review, Hussain et al. provided evidence for the therapeutic potential of strategies aimed at modulating aberrantly regulated processes and pathways in AD, HD and PD, such as protein aggregation, protein misfolding, inflammation, autophagy, glymphatic clearance, neurogenesis, glucose metabolism and the cholinergic system [4]. There have since been some exciting and positive achievements.

Indeed, at the beginning of 2020, Aducanumab (BIIB037), the monoclonal antibody developed by Biogen to reduce disease-specific protein aggregates in AD, has entered a Phase 3b open-label clinical trial with 2400 patients (ClinicalTrials.gov Identifier: NCT04241068) [9]. While the safety and tolerability outcomes are only expected in late 2023, Biogen has nevertheless submitted a biological license application with priority review for Aducanumab.

As for HD, the biggest therapeutic progress was observed in gene-based strategies aimed at directly reducing the expression of the mutant protein. Of these, the antisense oligonucleotide (ASO) Tominersen (IONIS-HTTRx), developed by Ionis Pharmaceuticals, showed a dose-dependent ability to reduce the mutant HD-causing protein in 34 HD patients participating in randomized, double-blind, multiple-ascending-dose Phase 1–2a trials [10]. In the spring of 2020, Roche announced that it had completed its enrolment of 791 HD patients for a Phase 3 multi-centered trial of Tominersen (ClinicalTrials.gov Identifier: NCT03761849) aimed at evaluating the safety and efficacy of the ASO over a two year period.

Conversely, therapeutic development for PD has seen less progress towards novel treatments that could potentially replace levodopa, which has well-described acute and chronic adverse effects [11]. However, in the late summer of 2019, the FDA approved istradefylline, developed by Kyowa Kirin, as a complementary PD drug that can be used when symptoms appear between regular levodopa doses [12].

There have thus been positive therapeutic advancements in the field of neurodegenerative diseases since Hussain et al. published their review approximately two years ago [4]. Not to be forgotten or dismissed are also the countless research and medical endeavors that have contributed to the culmination of these accomplishments, including the numerous experiments with negative outcomes that have paved the way for evolving knowledge, new insights and changing paradigms.

At the end of their review, Hussain et al. stated: “Therefore, despite substantial advances in the development of symptomatic treatments for neurodegenerative diseases, scientific efforts should not waiver, and perseverance is called for to attain this global goal” [4]. This ultimate goal being to significantly delay, if not prevent, disease progression and early death in patients with AD, PD, HD and other devastating neurodegenerative diseases. It is thus imperative that, amidst the recent positive therapeutic developments, scientific, clinical and pharmaceutical endeavors continue to pursue strategies such as improving delivery of ASOs with chemical modifications, bioconjugations and nanocarriers [13], repurposing drugs used in other conditions that target shared aberrant pathways [14,15] and considering the impact and therapeutic importance of non-neuronal tissues and organs [16,17,18].

Combining current effective approaches with additional relevant strategies that have proven beneficial in other conditions will most likely be the key to successfully achieving the goal of providing life-saving treatments to patients living with neurodegenerative diseases.
